# A multidimensional model of turnover intention in sport science academia

**DOI:** 10.3389/fpsyg.2026.1802362

**Published:** 2026-05-21

**Authors:** Osman Tolga Togo, Ali Gurel Goksel, Taner Yilmaz, Kaan Salman, İrem Kaptangil Çalışır, Mevlut Yildiz, Doğukan Batur Alp Gülşen

**Affiliations:** 1 Faculty of Sport Sciences, Marmara University, Istanbul, Türkiye; 2 Faculty of Sport Sciences, Muğla Sıtkı Koçman University, Muğla, Türkiye; 3 Faculty of Sport Sciences, Uşak University, Uşak, Türkiye; 4 Faculty of Sport Sciences, Van Yüzüncü Yıl University, Van, Türkiye; 5 Bitlis Eren University Social Sciences Vocational School, Bitlis Eren University, Bitlis, Türkiye; 6 Faculty of Sport Sciences, Aydın Adnan Menderes University, Aydın, Türkiye

**Keywords:** higher education environment, job satisfaction, organisational psychology, psychological safety, workplace dynamics

## Abstract

**Background:**

This study investigates the organisational predictors of turnover intention amongst higher education academics, particularly in sports sciences, where academics manage a unique dual role involving both theoretical teaching and practical sports lessons.

**Methods:**

The study explores how organisational identification, commitment, psychological safety, job satisfaction, and mental well-being contribute to turnover intention. Data were collected from 263 sports science academics using validated scales, and hypotheses were tested regarding these variables’ direct and indirect effects.

**Results:**

The results revealed that organisational commitment positively influences psychological safety and indirectly enhances job satisfaction and mental well-being, which reduces turnover intentions. However, the direct effects of organisational identification on psychological safety and turnover intention were insignificant. Psychological safety also positively impacted mental well-being and job satisfaction but did not directly affect turnover intention.

**Conclusion:**

These findings highlight the importance of fostering organisational commitment and psychological safety to enhance job satisfaction and well-being amongst sports science academics, who face unique challenges due to their dual roles. The study provides insights for university administrators and policymakers to develop targeted strategies that reduce turnover intentions by supporting a more positive and inclusive work environment.

## 1 Introduction

### 1.1 Organisational identification

Organisational identification (OI) reflects how closely individuals align their identity with their organisation, influencing outcomes such as job satisfaction, commitment, and performance. Rooted in social identity theory, OI suggests that individuals derive part of their self-concept from organisational membership (Wang et al., 2017). OI has been linked to enhanced employee engagement and positive behaviours like organisational citizenship behaviour (OCB) (Çek and Eyupoglu, 2019). Communication climate, leadership styles, and corporate social responsibility (CSR) initiatives can strengthen or weaken this bond (Bartels et al., 2010). In higher education institutions (HEIs), OI critically shapes the experiences of academic and administrative staff, affecting their commitment, particularly in environments with high turnover and talent competition (Al-Refaei et al., 2021). Effective communication, especially vertical communication, has enhanced OI, improving job satisfaction and commitment in HEIs (Bartels et al., 2010).

Moreover, training initiatives positively impact affective and normative commitment, suggesting that investing in professional development strengthens employees’ identification with the institution (Al-Refaei et al., 2021; Bashir and Long, 2015). Leadership also plays a key role, as leaders who embody institutional values can significantly enhance OI (Black, 2015). Additionally, CSR practises in HEIs positively influence OI, highlighting the importance of aligning institutional values with those of the employees to foster a cohesive organisational identity (Çek and Eyupoglu, 2019; Dzięgiel and Wojciechowska, 2017).

### 1.2 Organisational commitment

Based on Meyer and Allen’s model, organisational commitment (OC) reflects employees’ psychological attachment to their organisation and is often divided into affective, normative, and continuance commitment (Moyo, 2019). Affective commitment involves emotional attachment, normative commitment relates to a sense of obligation, and continuance commitment is based on the perceived costs of leaving (Fischer et al., 2020). These dimensions influence employee behaviour, performance, and retention, particularly in higher education.

OC is closely linked to organisational identification, with employees who strongly identify with their organisation likely to show higher commitment, enhancing performance and reducing turnover (Berberoğlu, 2015). In higher education institutions (HEIs), factors like job satisfaction, compensation, and professional development are significant in fostering OC amongst academic staff (Khan et al., 2014; Mabaso and Dlamini, 2018). For example, job satisfaction and OC directly influence the intention to leave, highlighting the importance of understanding faculty motivation (Khan et al., 2014).

The dynamics of OC in higher education are complex due to the unique challenges academic institutions face. Training and development positively impact affective and normative commitment, though they do not significantly affect continuance commitment (Al-Refaei et al., 2021). HEIs should, therefore, focus on training that strengthens emotional and normative ties. Transformational leadership is also crucial in promoting OC, as leaders who inspire and empower staff can significantly boost commitment and improve performance (Achmad and Mz, 2022; Mahfouz et al., 2020).

Organisational culture plays a vital role in OC within higher education. A supportive and inclusive culture fosters a sense of belonging, which enhances commitment (Masouleh and Allahyari, 2017). A positive culture is correlated with higher OC, influencing employee performance and satisfaction (Kayani, 2023). Additionally, work-life balance mediates the relationship between OC and employee performance, highlighting its importance (Saputra and Gorda, 2024).

### 1.3 Mental well-being

Mental well-being is a multifaceted concept that encompasses emotional, psychological, and social dimensions. It is characterised by the ability to cope with stress, work productively, and contribute to the community, as defined by the World Health Organization (WHO) (Agrawal and Sharma, 2022). The literature indicates that mental well-being is crucial for individuals, particularly during transitional life stages, such as adolescence and young adulthood, when mental health issues often emerge (Agrawal and Sharma, 2022). Various studies have highlighted the importance of coping mechanisms and social support in maintaining mental well-being, especially in stressful environments (Schmidt and Hansson, 2018; Küçükalpelli et al., 2025; Kesler et al., 2026). For instance, Schmidt and Hansson (2018) emphasise that coping strategies are essential for doctoral students to manage stress and maintain their mental health (Schmidt and Hansson, 2018).

Furthermore, the COVID-19 pandemic has exacerbated mental health challenges globally, leading to increased anxiety and depression amongst various populations, including students (Kaur et al., 2022; Son et al., 2020). In the context of higher education, mental well-being becomes even more critical due to the unique stressors faced by students. The transition to university life often involves significant changes, including relocation, academic pressures, and social isolation, which can adversely affect mental health (Kaur et al., 2022; Son et al., 2020). Research indicates that the pandemic has led to a notable decline in mental health amongst college students, with many reporting increased feelings of anxiety and depression (Kaur et al., 2022; Son et al., 2020; Islam et al., 2020). For example, a study conducted in the United States found that the pandemic significantly impacted students’ mental health, highlighting the urgent need for mental health support services (Son et al., 2020).

Additionally, findings reveal that the relocation from campus during the pandemic heightened stress and feelings of isolation, further deteriorating students’ mental well-being (Kaur et al., 2022). Higher education institutions play a pivotal role in supporting students’ mental health. Robson (2021) discusses how these institutions can serve as mechanisms to enhance mental well-being by providing resources and support systems (Robson, 2021). Initiatives to improve mental health literacy and access to mental health services are essential for fostering an environment conducive to well-being (Agrawal and Sharma, 2022; Siddique et al., 2022). Moreover, studies have shown that students who engage in part-time distance education often report better mental health outcomes, suggesting that flexible learning options can be beneficial (Robson, 2021). However, despite the increasing awareness of mental health issues, many students still do not seek help due to stigma or lack of knowledge about available resources (Gorczynski et al., 2017; Sojindamanee et al., 2023). The literature also highlights the disparities in mental health outcomes amongst different student populations. For instance, a study in Bangladesh found that over half of the university students surveyed reported poor mental health, indicating a significant public health concern (Ovi et al., 2024). Similarly, the prevalence of mental health disorders amongst university students in various countries underscores the need for targeted interventions (Getachew and Tekle, 2018; Asghari et al., 2022). Furthermore, peer relationships and social support networks are crucial in mitigating mental health challenges during stressful periods, such as the COVID-19 pandemic (Conceição et al., 2021).

### 1.4 Job satisfaction

Job satisfaction, a key area in organisational studies, is closely linked to organisational identification—the extent to which individuals align with their organisation’s values and goals. This review explores the relationship between organisational identification and job satisfaction, particularly in higher education institutions. Research suggests that organisational identification acts as a mediator between various organisational dynamics and job satisfaction. For example, Loi et al. (2013) found that leader-member exchange (LMX) quality influences job satisfaction through organisational identification, indicating that solid identification enhances satisfaction by providing support and resources (Loi et al., 2013). Similarly, Ashforth et al. (2013) highlighted a positive correlation between organisational identification and job satisfaction, emphasising its role as a mediator for organisational outcomes (Ashforth et al., 2013). Sökmen (2019) also established that a strong sense of belonging positively affects organisational commitment and job satisfaction. The relationship between organisational identification and job satisfaction has unique characteristics in higher education institutions (Sökmen, 2019). Coskuner et al. (2018) showed that solid organisational identification can mitigate negative experiences like mobbing and improve job satisfaction (Coskuner et al., 2018). Wilkins et al. (2016) found that organisational identification influences student commitment and satisfaction, suggesting that its principles apply beyond traditional employment relationships (Wilkins et al., 2016). Yılmaz and Kaya (2022) demonstrated that teachers’ perceptions of organisational identification positively impact their job satisfaction and professional engagement (Yılmaz and Kaya, 2022). The role of organisational culture and leadership in shaping job satisfaction through organisational identification is also crucial. Yusuf (2019) indicated that a supportive organisational culture enhances job satisfaction amongst lecturers by aligning with their needs (Yusuf, 2019). Mihci and Uzun (2020) found that ethical leadership improves organisational justice and identification perceptions, leading to higher job satisfaction (Mihci and Uzun, 2020).

### 1.5 Psychological safety

Psychological safety, a shared belief that the team is safe for interpersonal risk-taking, has garnered significant attention in organisational psychology. It is crucial for fostering an environment where individuals feel secure to express their thoughts, concerns, and ideas without fear of negative consequences. This construct has been extensively studied across various organisational contexts, revealing its implications for team dynamics, innovation, and overall performance (Edmondson and Lei, 2014; Soares and Lopes, 2014; Schulte et al., 2012). Psychological safety is deeply intertwined with organisational identification, which refers to how individuals align their identity with the organisation’s. This alignment can enhance feelings of belonging and commitment, fostering a psychological safety culture (Wilkins et al., 2016; Kaya et al., 2017). In higher education institutions (HEIs), the relevance of psychological safety is particularly pronounced. The educational environment is inherently complex, with diverse interactions amongst students, faculty, and administrative staff. Research indicates that psychological safety in HEIs contributes to individual well-being and enhances collective learning and performance (Sher et al., 2019; Soares and Lopes, 2017). For instance, it is emphasised that understanding the antecedents of psychological safety within HEIs is critical for developing policies that promote a supportive atmosphere, ultimately leading to improved organisational performance and employee satisfaction (Sher et al., 2019).

Furthermore, the role of authentic leadership in fostering psychological safety has been highlighted, suggesting that leaders who model openness and trust can significantly impact the psychological climate of educational settings (Soares and Lopes, 2017). Moreover, the interplay between psychological safety and organisational identification in higher education is noteworthy. Studies have shown that when students and staff identify strongly with their institution, they are more likely to experience a sense of psychological safety, leading to greater academic engagement and satisfaction (Wilkins et al., 2016).

### 1.6 Turnover intention

Turnover intention, or the desire to leave one’s current role, is crucial in organisational behaviour. It is influenced by factors like organisational identification, where employees align with their organisation’s values and goals. This alignment often leads to more significant commitment and reduced turnover intention. Research by Aitken and von Treuer (2020) shows that organisational identification overlaps with affective commitment, meaning employees who strongly identify with their organisation are less likely to want to leave (Aitken and von Treuer, 2020). Supportive environments, highlighted by perceived organisational support (POS) and perceived supervisor support (PSS), also correlate with lower turnover intentions (Kalidass and Bahron, 2015).

In higher education institutions, turnover intention is notably affected by job satisfaction, organisational culture, and career development opportunities. Dewi and Nurhayati (2021) found that investing in employees’ career growth reduces turnover rates by strengthening organisational commitment (Dewi and Nurhayati, 2021). Additionally, as Zhang and Feng (2011) note, job satisfaction negatively correlates with academic staff turnover intention (Zhang and Feng, 2011).

Leadership styles significantly impact turnover intention, with abusive supervision linked to higher turnover, particularly in stressful environments like education (Lyu et al., 2019). Engaged employees who feel supported and satisfied are less likely to consider leaving (Zahari et al., 2020).

### 1.7 The present study

This study investigates the organisational predictors of turnover intention amongst higher-education academics, focusing on the direct and indirect effects of critical variables such as organisational identification, organisational commitment, psychological safety, job satisfaction, and mental well-being. Given the ongoing challenges in retaining academic staff, understanding these predictors is critical for fostering a sustainable and supportive work environment in higher education institutions.

In the field of sports sciences, academics hold a unique position that differentiates them from other disciplines. They are responsible for delivering theoretical courses and teaching practical sports lessons, a dual role that requires balancing diverse pedagogical approaches and addressing various physical and cognitive demands. This dual responsibility may place additional pressure on these academics, highlighting the relevance of psychological factors such as psychological safety, job satisfaction, and mental well-being.

The psychological variables under investigation in this study are particularly pertinent for sports science academics because they directly influence their ability to thrive in their multifaceted roles. High levels of psychological safety, for example, encourage them to engage more openly in interdisciplinary collaboration and innovative teaching methods. At the same time, organisational identification and commitment can foster a sense of belonging and purpose, which is crucial given their dynamic and often challenging work environment.

The study is structured to test hypotheses that elucidate the complex dynamics leading to turnover intention. It proposes several direct effects, such as the influence of organisational identification, organisational commitment, psychological safety, job satisfaction, and mental well-being on turnover intention (H1–H5). For instance, it is hypothesised that organisational identification and organisational commitment will directly reduce turnover intention. At the same time, psychological safety, job satisfaction, and mental well-being will also have direct adverse effects on turnover intention.

Additionally, the study explores indirect pathways through which these variables may influence turnover intention. For example, it examines how organisational identification may indirectly predict turnover intention through its effects on mental well-being, psychological safety, and job satisfaction (H1a–H1c). Similarly, it investigates how organisational commitment indirectly impacts turnover intention via psychological safety, job satisfaction, and mental well-being (H2a–H2c). The study also considers the indirect effects of Psychological Safety on turnover intention through mental well-being and job satisfaction (H3a–H3b).

By testing these direct and indirect pathways, the present study aims to provide a more comprehensive understanding of how organisational dynamics contribute to academic turnover intentions. The findings are expected to have practical implications for university administrators and policymakers in designing strategies that enhance organisational identification, organisational commitment, and psychological safety, ultimately promoting job satisfaction and mental well-being and reducing turnover intentions in higher education settings.

Although constructs such as Organisational Identification (OI) and Organisational Commitment (OC) appear conceptually related, they represent distinct psychological mechanisms within this model. Whilst OI reflects a cognitive and self-definitional alignment with the institution, OC captures a more functional and affective bond directly linked to behavioural outcomes. This study justifies their simultaneous inclusion by aiming to disentangle the relative predictive power of identity-based versus commitment-based mechanisms on turnover intention, thereby providing a more theoretically parsimonious yet comprehensive understanding of academic withdrawal.

Beyond examining direct and indirect relationships, this study seeks to clarify the relative contribution of identity-based (organisational identification) and commitment-based (organisational commitment) mechanisms in predicting turnover intention within sport sciences academia. Although these constructs are often treated as conceptually overlapping, their differential roles in explaining withdrawal-related outcomes remain underexplored, particularly in higher education contexts. By disentangling these mechanisms within a single structural framework, the present study aims to refine theoretical understanding of turnover processes in academic environments.

*H1*: Organisational Identification negatively and directly predicts Turnover Intention.

*H1a*: Organisational Identification positively and indirectly predicts Turnover Intention through Mental Well-Being.

*H1b*: Organisational Identification positively and indirectly predicts Turnover Intention through Psychological Safety.

*H1c*: Organisational Identification positively and indirectly predicts Turnover Intention through Job Satisfaction.

*H2*: Organisational Commitment negatively and directly predicts Turnover Intention.

*H2a*: Organisational Commitment positively and indirectly predicts Turnover Intention through Psychological Safety.

*H2b*: Organisational Commitment positively and indirectly predicts Turnover Intention through Job Satisfaction.

*H2c*: Organisational Commitment positively and indirectly predicts Turnover Intention through Mental Well-Being.

*H3*: Psychological Safety negatively and directly predicts Turnover Intention.

*H3a*: Psychological Safety positively and indirectly predicts Turnover Intention through Mental Well-Being.

*H3b*: Psychological Safety positively and indirectly predicts Turnover Intention through Job Satisfaction.

*H4*: Job Satisfaction negatively and directly predicts Turnover Intention.

*H5*: Mental Well-Being negatively and directly predicts Turnover Intention.

## 2 Methods

The present study was grounded in a theory-driven structural research model and tested using structural equation modelling (SEM) to examine the multidimensional mechanisms underlying turnover intention in sport science academia. SEM was selected as it allows for the simultaneous estimation of multiple direct and indirect relationships amongst latent constructs, whilst accounting for measurement error (Kline, 2016; Hair et al., 2022).

The hypothesised model was tested using SEM to evaluate the overall model fit and to estimate direct, indirect, and total effects amongst the latent variables. This approach enables a comprehensive examination of the multidimensional and process-oriented nature of turnover intention in academic contexts (Hair et al., 2022).

### 2.1 Participant

Our study, which involved 263 sports science academics (*M*_age_ = 38.59 ± 9.06), is unique in its approach. Notably, 33.5% of the participants were female (*n* = 88) and 66.5% were male (*n* = 175). Participants were recruited using a convenience sampling strategy. The participants represented a diverse range of academic ranks, with 12.5% being Prof. Dr. (*n* = 33), 26.6% Assoc. Prof. Dr. (*n* = 70), 22.1% Assist. Prof. Dr. (*n* = 58), 7.2% lecturer (*n* = 19), 5.3% Lecturer with PhD (*n* = 14), 5.3% Research Assistant with PhD (*n* = 14), and 20.9% Research Assistant (*n* = 55). Regarding administrative roles, 69.6% of participants did not hold administrative positions (*n* = 183), whilst 30.4% had administrative responsibilities (*n* = 80). Most participants, 95.4%, worked in public institutions (*n* = 251) and 4.6% in private institutions (*n* = 12). Our findings also shed light on tenure expectations, with 75.7% not expecting a position (*n* = 199) and 24.3% waiting for a position (*n* = 64). A significant finding was that 69.2% of the participants felt their salary did not reflect their efforts (*n* = 182), whilst 30.8% felt it did (*n* = 81). The participants had an average of 10.02 years (±9.018) of experience in their academic careers and 7.02 years (±6.701) of experience at their current institutions. In terms of publications, participants reported an average of 1.38 (±2.410) publications in Q1 journals.

Participants were recruited using a convenience sampling strategy targeting academics working in sport science faculties at public universities in Türkiye. Invitations to participate in the study were distributed via institutional email networks and professional academic communication channels. To be included in the study, participants had to be actively employed as academic staff in a sport sciences faculty and voluntarily agree to participate in the survey. Responses from individuals not currently holding an academic position or providing incomplete questionnaires were excluded from the analysis.

### 2.2 Data collection instruments

All constructs were measured using previously validated scales widely used in organisational behaviour research. Where necessary, scales were adapted to the academic context of sport sciences. Internal consistency reliability was assessed using Cronbach’s alpha, and the structural relationships amongst the constructs were examined using structural equation modelling.

#### 2.2.1 Organisational identification

Organisational identification was assessed with a 6-item scale developed by Mael and Ashforth (1992) and adapted to Turkish by Melikoğlu (2009), Mael and Ashforth (1992), and Melikoğlu (2009). The internal consistency coefficient for the Turkish version of the scale was found to be 0.81. The internal consistency coefficient in this study is 0.89.

#### 2.2.2 Organisational commitment

The KUT scale is a four-item, one-dimensional scale that is used by rating it on a 5- or 7-point scale. This scale includes four items created per the Klein et al. (2012) definition of commitment. The first item expresses the general structural feature, the second expresses dedication to the goal, the third represents the will or will for commitment, and the last expresses responsibility towards the goal. Klein et al. (2014) tested the structure of the scale in five different sample groups by collecting data from 2,487 participants from various environments, professions, organisations, and industries. The Turkish scale translation was made by Şenel et al. (2020).

#### 2.2.3 Mental well-being

The Warwick-Edinburgh Mental Well-being Scale Short Form, developed by Tennant et al. (2007) and translated into Turkish by Demirtaş and Baytemir (2019), evaluates mental well-being. It is a 7-item scale with positive statements. When responding, participants are asked to consider their experiences over the past 2 weeks.

#### 2.2.4 Job satisfaction

Job satisfaction was measured using the Job Satisfaction Subscale of the Michigan Organisational Assessment Survey, which consists of 3 questions. The scale was developed by Cammann et al. (1983) and adapted to Turkish by Çevirgen and Üngüren (2009). The internal consistency coefficient of the Turkish form of the scale was found to be 0.65. The internal consistency coefficient in this study is 0.76.

#### 2.2.5 Psychological safety

The Psychological Safety Scale, consisting of 7 items, assesses psychological safety in institutions. The scale was developed by Edmondson (1999) and adapted to Turkish by Yener (2015). The scale includes items such as “It is safe to take risks on this team”; “If I make a mistake on this team, it will not be used against me.”

#### 2.2.6 Turnover intention

Turnover Intention was measured with a 3-item scale developed by Wayne et al. (1997) and adapted to Turkish by Küçükusta (2007). The internal consistency coefficient for the Turkish form of the scale was found to be 0.69. The internal consistency coefficient in this study is 0.92.

### 2.3 Process

Before the research process began, approval was obtained from the relevant ethics committee. Ethics committee approval ensured that the research complied with scientific and ethical standards. Informed consent was obtained from the participants before they participated in the study. The purpose of the research, procedures, potential risks, and voluntary participation were explained in detail in the consent form. An online form was used for the data collection process. The online form was designed to ensure participants could easily access it and submit their responses digitally. The online form was sent to relevant sports science academics via social media. The post’s content included the link to the form and the necessary instructions for filling it out. Participants completed the questionnaire online. The form was anonymous, and no identifying information was collected. Data collection was completed within the specified period whilst ensuring participants’ confidentiality.

### 2.4 Analysis

Prior to conducting the structural equation modelling, the dataset was screened for potential violations of statistical assumptions. Descriptive statistics, skewness, and kurtosis values were examined to assess the normality of the variables. The values were within acceptable ranges, indicating that the data were suitable for SEM analysis. Subsequently, the hypothesised structural model was tested using AMOS software to estimate both direct and indirect relationships amongst the latent variables.

For this study, we used AMOS software to analyse the proposed model through structural equation modelling (SEM). The analysis was conducted in several stages to ensure accuracy and comprehensiveness. First, we prepared the data for analysis. This involved cleaning the dataset to address missing values, outliers, or inconsistencies. We also calculated descriptive statistics to understand the sample’s characteristics and the distribution of each variable. Next, we specified the hypothesised model in AMOS. The model included Organisational Identification (OI) and Organisational Commitment (OC) as predictors, Mental Well-Being (MWB) as a mediator, and Psychological Safety (PS) and Job Satisfaction (JS) as additional mediators. Turnover Intention (TI) was the outcome variable. We analysed the structural model to estimate the direct and indirect relationships amongst the variables as specified in the hypothesised model. To evaluate the fit of the model, we used several fit indices. These included the Chi-Square Test to assess the overall fit, the Comparative Fit Index (CFI) and the Tucker-Lewis Index (TLI) to evaluate comparative and relative fit, and the Root Mean Square Error of Approximation (RMSEA) to measure the approximation error. We interpreted the results by examining both the direct and indirect effects. This helps to understand how the predictors (OI and OC) influence Turnover Intention (TI) through the mediators (MWB, PS, and JS). Mediation analysis provided insights into the role of MWB, PS, and JS in the relationships amongst the predictors and the outcome.

All constructs included in the study were measured using previously validated scales that have been widely applied in organisational behaviour and higher education research. Where necessary, these scales were adapted to the context of sport science academia based on previously validated Turkish versions. Internal consistency reliability was assessed using Cronbach’s alpha coefficients. Structural equation modelling (SEM) was performed using AMOS software, which enables the simultaneous evaluation of relationships amongst latent variables whilst accounting for measurement error. Model fit was evaluated using commonly recommended indices, including χ^2^/df, CFI, TLI, and RMSEA. Data were collected from a single source via self-reports, raising concerns regarding Common Method Bias (CMB). To address this, Harman’s single-factor test was performed, and the results indicated that no single factor accounted for the majority of the variance, suggesting that CMB does not significantly threaten the validity of our findings. However, the lack of a marker variable or CFA-based statistical controls remains a limitation. The measurement model was evaluated using confirmatory factor analysis (CFA) to verify the structural validity of the constructs. All items were constrained to load on their respective latent factors.

## 3 Results

Organisational Identification was relatively high (*M* = 30.12 ± 9.11), suggesting a strong sense of identification with the organisation amongst the participants. Turnover Intention was moderate (*M* = 6.53 ± 4.98), indicating a noticeable intention to leave the organisation. Job Satisfaction was relatively high (*M* = 16.10 ± 3.85), reflecting overall satisfaction with the job. Organisational commitment was also high (*M* = 21.33 ± 5.81), suggesting a solid commitment to the organisation. Psychological safety was moderately high (*M* = 27.96 ± 8.65), indicating a generally safe environment for expressing concerns. Finally, Mental Well-Being was also moderately high (*M* = 36.83 ± 8.03), reflecting good overall mental health (Table 1).

**Table 1:** Descriptive statistics, skewness–kurtosis values, and correlations amongst study variables.

Variable	M ± SD	Skew.	Kurt.	α	1	2	3	4	5
1. Organisational identification	30.12 ± 9.11	−0.75	−0.10	0.89	1				
2. Turnover intention	6.53 ± 4.98	1.42	1.08	0.90	−0.27^**^	1			
3. Job Satisfaction	16.10 ± 3.85	−0.72	−0.23	0.69	0.48^**^	−0.66^**^	1		
4. Organisational commitment	21.33 ± 5.81	−1.08	0.86	0.95	0.72^**^	−0.45^**^	0.64^**^	1	
5. Psychological safety	27.96 ± 8.65	−0.22	−0.27	0.82	0.32^**^	−0.39^**^	0.51^**^	0.43^**^	1
6. Mental well-being	36.83 ± 8.03	−0.81	0.87	0.88	0.52^**^	−0.48^**^	0.65^**^	0.57^**^	0.50^**^

**p < 0.01.

Correlations amongst the variables were generally significant, indicating several meaningful relationships. Specifically, Organisational Identification was positively correlated with Job Satisfaction (*r* = 0.48, *p* < 0.01), Organisational Commitment (*r* = 0.72, *p* < 0.01), Psychological Safety (*r* = 0.32, *p* < 0.01), and Mental Well-Being (*r* = 0.52, *p* < 0.01). Turnover Intention was negatively correlated with Job Satisfaction (*r* = −0.66, *p* < 0.01), Organisational Commitment (*r* = −0.45, *p* < 0.01), Psychological Safety (*r* = −0.39, *p* < 0.01), and Mental Well-Being (*r* = −0.48, *p* < 0.01). Job Satisfaction was positively correlated with Organisational Commitment (*r* = 0.64, *p* < 0.01), Psychological Safety (*r* = 0.51, *p* < 0.01), and Mental Well-Being (*r* = 0.65, *p* < 0.01). Organisational Commitment was positively correlated with Psychological Safety (*r* = 0.43, *p* < 0.01) and Mental Well-Being (*r* = 0.57, *p* < 0.01). Psychological safety was positively correlated with Mental Well-Being (*r* = 0.50, *p* < 0.01).

### 3.1 Hypothesised model

The results of the measurement model indicated a satisfactory fit to the data: *x*^2^/df = 2.45, CFI = 0.93, TLI = 0.92, and RMSEA = 0.07. All factor loadings were statistically significant (*p* < 0.001) and ranged from 0.52 to 0.94, exceeding the recommended threshold of 0.50.

**Figure 1:**
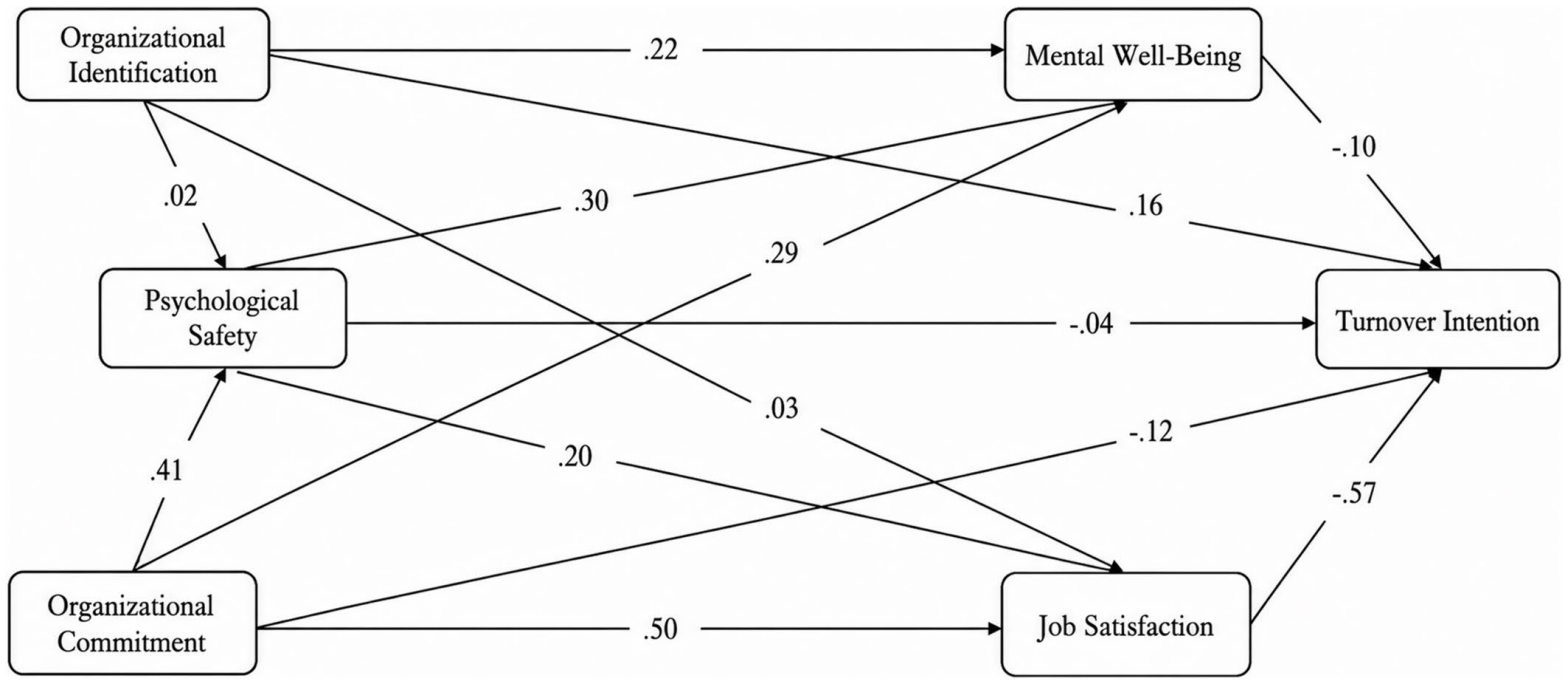
Structural equation model illustrating the direct and indirect effects of organisational identification, psychological safety, and organisational commitment on mental well-being, job satisfaction, and turnover intention.

The findings of this study provide nuanced insights into the relationships between Organisational Identification, Organisational Commitment, Psychological Safety, Job Satisfaction, Mental Well-Being, and Turnover Intention amongst academics in higher education (Figure 1). The direct effect of Organisational Identification on Psychological Safety was insignificant (*β* = 0.018, *p* > 0.05), leading to the rejection of H1. However, the impact of Organisational Commitment on Psychological Safety was positive and significant (*β* = 0.414, *p* < 0.05), supporting H2. The effect of Psychological Safety on Mental Well-Being was positive and significant (*β* = 0.304, *p* < 0.05), confirming H3. Additionally, the effect of Psychological Safety on Job Satisfaction was also found to be positive and significant (*β* = 0.289, *p* < 0.05), supporting H4. The direct effect of Organisational Identification on Mental Well-Being was positive and significant (*β* = 0.216, *p* < 0.05), leading to the acceptance of H5. However, the direct effect of Organisational Identification on Job Satisfaction was insignificant (*β* = 0.029, *p* > 0.05), resulting in rejecting H1a. The effect of Organisational Commitment on Job Satisfaction was positive and significant (*β* = 0.496, *p* < 0.05), confirming H6. Similarly, the effect of Organisational Commitment on Mental Well-Being was also positive and significant (*β* = 0.288, *p* < 0.05), leading to the acceptance of H7. The effect of Job Satisfaction on Turnover Intentions was negative and significant (*β* = −0.569, *p* < 0.05), supporting H8. However, the effect of Psychological Safety on Turnover Intention was negative but insignificant (*β* = −0.044, *p* > 0.05), leading to the rejection of H9. Similarly, the effect of Mental Well-Being on Turnover Intention was negative but insignificant (*β* = −0.104, *p* > 0.05), resulting in the rejection of H10. The effect of Organisational Commitment on Turnover Intention was also negative but insignificant (*β* = −0.118, *p* > 0.05), leading to the rejection of H11. Furthermore, the effect of Organisational Identification on Turnover Intention was positive but insignificant (*β* = 0.157, *p* > 0.05), resulting in the rejection of H12.

Regarding indirect effects, Organisational Commitment had a significant indirect impact on Job Satisfaction (*β* = 0.120, *p* < 0.05), supporting H2a, and a significant indirect impact on Mental Well-Being (*β* = 0.126, *p* < 0.05), confirming H2b. Additionally, the indirect effect of Organisational Commitment on Turnover Intention was negative and significant (*β* = −0.412, *p* < 0.05), supporting H2c. In contrast, the indirect impact of Organisational Identification on Job Satisfaction and Mental Well-Being was insignificant (*β* = 0.005 and *β* = 0.006, respectively, *p* > 0.05), leading to the rejection of H1a and H1b. The indirect effect of Organisational Identification on Turnover Intention was also insignificant (*β* = −0.043, *p* > 0.05), resulting in the rejection of H1c. The indirect impact of Psychological Safety on other variables was not significant (*β* = −0.196, *p* > 0.05), leading to the rejection of H3a, H3b, and H3c.

## 4 Discussion

Analysing the relationships amongst Organisational Identification, Organisational Commitment, Psychological Safety, Job Satisfaction, Mental Well-Being, and Turnover Intention within higher education in sports sciences reveals a complex and multifaceted interplay amongst these constructs. The findings indicate that Organisational Identification, often viewed as a foundational element in fostering a positive organisational environment, does not directly impact Turnover Intention or Psychological Safety, suggesting that its influence may be more limited than previously assumed. This is particularly noteworthy given that previous research has established a strong link between organisational identification and various positive outcomes, including employee engagement and performance (Lee et al., 2015). The current study suggests that Psychological Safety may be more contingent upon specific organisational behaviours and practises, such as those associated with Organisational Commitment, rather than merely an employee’s identification. This aligns with the notion that interpersonal dynamics and organisational culture play a critical role in shaping an individual’s sense of safety and security in the workplace (Frazier et al., 2017).

The significant positive relationship between Organisational Commitment and Psychological Safety (*β* = 0.414, *p* < 0.05) reinforces the idea that employees who are deeply committed to their organisation are more likely to feel secure in voicing their concerns and ideas (H2, accepted). This finding is consistent with existing literature that emphasises the importance of commitment in creating a supportive environment where employees feel psychologically safe (Liu et al., 2021). Furthermore, the positive effects of Psychological Safety on both Mental Well-Being and Job Satisfaction underscore its pivotal role in fostering a healthy work environment. When employees perceive that they can express themselves without fear of negative consequences, their mental health and job satisfaction are likely to improve, which is particularly salient in high-stress fields such as sports sciences (Choori, 2023).

Interestingly, whilst Organisational Identification positively influences Mental Well-Being, its non-significant effect on Job Satisfaction raises questions about the nature of these constructs (H1a, rejected). This suggests that factors contributing to job satisfaction may be more closely tied to specific job-related elements rather than an employee’s identification with the organisation. This finding is echoed in the literature, which indicates that job satisfaction is often influenced by tangible aspects of the job, such as workload, compensation, and work-life balance, rather than abstract notions of identification (Urbonas et al., 2015). The significant positive effects of Organisational Commitment on both Job Satisfaction and Mental Well-Being (*β* = 0.288, *p* < 0.05) further highlight the importance of fostering a strong sense of commitment amongst employees. This aligns with previous studies that have found a direct link between organisational commitment and positive employee outcomes, including job satisfaction and reduced turnover intention (Steffens et al., 2017). The negative relationship between Job Satisfaction and Turnover Intention is also consistent with expectations, as higher job satisfaction typically correlates with lower intentions to leave the organisation. This finding underscores the necessity of enhancing job satisfaction as a strategy for employee retention, particularly in the competitive field of higher education (Jing et al., 2011). The non-significant direct effect of Organisational Identification on Job Satisfaction and Turnover Intention contrasts with prior findings suggesting a stronger association between identification and withdrawal-related outcomes (Lee et al., 2015; Urbonas et al., 2015). Whilst organisational identification reflects cognitive alignment with the institution, turnover intention represents a behavioural orientation that may be more directly shaped by commitment-based and satisfaction-related mechanisms. In the present model, identification was associated with mental well-being but did not directly predict job satisfaction or turnover intention, indicating that identity perceptions alone may not be sufficient to influence withdrawal-related decisions within this context.

Interestingly, the non-significant direct effect of psychological safety on turnover intention suggests a complex mechanism in academic settings. Whilst a safe interpersonal climate is essential for well-being and satisfaction, it may not function as a direct deterrent to leaving the organisation. Within sport science faculties, psychological safety appears to operate as a distal factor that requires translation into proximal evaluative attitudes, such as job satisfaction, to influence withdrawal decisions. This finding indicates that even when academics feel safe to take interpersonal risks, structural or professional dissatisfactions may still drive turnover intentions if the safe environment does not lead to a tangible sense of satisfaction with one’s work conditions. Therefore, psychological safety is a necessary but not sufficient condition for retention in this specific academic context.

The indirect effects of Organisational Commitment on Job Satisfaction, Mental Well-Being, and Turnover Intention further elucidate its critical role in the organisational framework. The significant indirect impact on job satisfaction and mental well-being, together with the negative indirect effect on turnover intention, suggests that organisational commitment catalyses improvements in broader employee outcomes. This reinforces the need for organisations to cultivate commitment through supportive practises and policies (Islam et al., 2019).

In contrast, the non-significant indirect effects of Organisational Identification on Job Satisfaction, Mental Well-Being, and Turnover Intention indicate that its influence did not operate through the expected mediating pathways within the present model. Although identification was associated with mental well-being, it did not significantly predict psychological safety or job satisfaction, thereby limiting its indirect effect on turnover intention. This pattern suggests that identification may not directly translate into withdrawal-related consequences through these mechanisms within this framework (Deng et al., 2018).

Similarly, the insignificant indirect effects of Psychological Safety on Turnover Intention suggest that its influence may operate primarily at the attitudinal level rather than directly shaping withdrawal-related decisions. Consistent with prior literature (Edmondson and Lei, 2014; Frazier et al., 2017), psychological safety is often associated with learning behaviour, engagement, and well-being outcomes. In the present model, psychological safety significantly predicted mental well-being and job satisfaction; however, these pathways were insufficient to produce a statistically significant indirect effect on turnover intention. This finding indicates that whilst a psychologically safe climate may enhance positive work experiences, satisfaction-related and commitment-based mechanisms may play a more decisive role in shaping withdrawal intentions within academic contexts.

The findings of this study contribute to a deeper understanding of the dynamics between Organisational Identification, Organisational Commitment, Psychological Safety, Job Satisfaction, Mental Well-Being, and Turnover Intention. They reveal that whilst Organisational Identification is essential, its direct effects may be limited and that Organisational Commitment plays a more substantial role in influencing Psychological Safety and subsequent employee outcomes. The insights gained from this research underscore the necessity for higher education institutions, particularly in sports sciences, to prioritise the development of a committed workforce and to foster an environment where Psychological Safety is paramount. This approach will likely enhance employee satisfaction and well-being, organisational effectiveness, and retention rates.

Organisational identification, organisational commitment, psychological safety, job satisfaction, and mental well-being are critical constructs that significantly influence turnover intention. Understanding the interplay amongst these variables is essential for fostering a healthy organisational environment and mitigating turnover rates. This literature review synthesises existing research to elucidate these constructs’ importance and potential effects on turnover intention.

Organisational identification refers to the degree to which employees perceive themselves as part of their organisation, which can significantly affect their attitudes and behaviours. Lee et al. (2015) highlight that strong organisational identification can lead to positive work-related behaviours, such as increased job performance and reduced turnover intention (Lee et al., 2015). This is particularly relevant when employees feel a sense of belonging and alignment with the organisation’s goals. Furthermore, organisational identification has been shown to mediate the relationship between various factors, including perceived external prestige and turnover intention, suggesting that employees who identify strongly with their organisation are less likely to leave (Zhu et al., 2021).

Organisational commitment, closely related to identification, encompasses employees’ emotional attachment and loyalty towards their organisation. Research indicates that higher levels of organisational commitment correlate with lower turnover intentions (Gim et al., 2015). For instance, Gim et al. (2015) found that affective commitment negatively relates to turnover intention, reinforcing that emotionally invested employees are less likely to consider leaving. This emotional bond can be cultivated through supportive leadership and a positive organisational culture, enhancing job satisfaction and reducing turnover intentions (Tolksdorf et al., 2022).

Psychological safety, a shared belief that the team is safe for interpersonal risk-taking, plays a pivotal role in employee retention. Hebles et al. (2022) asserts that psychological safety fosters an environment where employees feel comfortable expressing their thoughts and concerns without fear of negative consequences (Hebles et al., 2022). This environment enhances job satisfaction and mitigates turnover intentions, particularly in high-stress contexts such as healthcare during the COVID-19 pandemic (Hebles et al., 2022). Psychological safety allows employees to engage more fully with their work, leading to more outstanding commitment and a reduced likelihood of leaving the organisation (Sobaih et al., 2022). Job satisfaction is another critical factor influencing turnover intention. Ding and Wu (2023) demonstrate that job satisfaction mediates the relationship between psychological empowerment and turnover intention, indicating that empowered employees who are satisfied with their jobs are less likely to leave (Ding and Wu, 2023).

Moreover, job satisfaction is often linked to various organisational outcomes, including productivity and employee engagement. The literature consistently shows that when employees are satisfied with their jobs, they are less likely to exhibit turnover intentions, contributing to a more stable workforce (Karimi et al., 2022). Mental well-being, encompassing psychological health and emotional stability, is crucial for employee retention. Selem et al. (2022) emphasise that positive psychological outcomes, such as mental well-being, can enhance employee resilience, thereby reducing turnover intentions (Selem et al., 2022). In high-stress environments, such as healthcare, the mental health of employees directly correlates with their intention to stay or leave. Studies indicate that when employees perceive adequate support for their mental well-being, they are less likely to consider leaving their jobs (Yang et al., 2021). The interplay between these constructs is vital for creating a healthy organisational environment. A culture that promotes organisational identification, commitment, psychological safety, job satisfaction, and mental well-being can lead to lower turnover intentions and a more engaged workforce. For instance, Kirk-Brown and van Dijk (2015) found that job resources and psychological safety significantly influence affective commitment, affecting turnover intentions (Kirk-Brown and van Dijk, 2015). This suggests that organisations should focus on enhancing job resources and fostering psychological safety to cultivate commitment and reduce turnover. Research has demonstrated that organisations with a healthy work environment significantly contribute to the well-being of their employees. A healthy organisation is characterised by practises that foster a positive organisational culture, including supportive leadership, clear communication, equitable policies, and opportunities for professional growth. Such environments enhance employees’ physical and psychological health and promote job satisfaction, engagement, and organisational commitment. By prioritising the well-being of their workforce, healthy organisations create a virtuous cycle where employee satisfaction and productivity reinforce one another, ultimately leading to sustainable organisational success (Gil-Lacruz et al., 2023; Segura-Camacho et al., 2018).

Moreover, the implications of these constructs extend beyond individual employees to the organisation as a whole. High turnover rates can lead to increased recruitment and training costs, loss of institutional knowledge, and decreased morale amongst remaining employees. Therefore, organisations that prioritise the well-being of their employees and foster a culture of identification and commitment are likely to experience enhanced performance and reduced turnover (Amponsah-Tawiah et al., 2016).

Beyond testing a comprehensive structural model, this study contributes theoretically by differentiating the relative influence of identity-based and commitment-based mechanisms in predicting turnover intention within sport sciences academia. Whilst organisational identification is frequently associated with positive organisational outcomes in prior research, the present findings suggest that behavioural attachment in the form of organisational commitment may operate as a more functionally proximal mechanism in explaining turnover intention. This distinction refines existing theoretical models by indicating that cognitive alignment with the organisation (identification) does not necessarily translate into withdrawal-related outcomes when not accompanied by stronger commitment-based bonds. Additionally, the findings suggest that psychological safety may function more robustly as a well-being and satisfaction enhancer rather than as a direct predictor of turnover intention within this academic context.

## 5 Limitations

Whilst this study provides valuable insights into the relationships amongst Organisational Identification, Organisational Commitment, Psychological Safety, Job Satisfaction, Mental Well-Being, and Turnover Intention amongst sports science academics, several limitations must be acknowledged. First, the study’s cross-sectional design precludes the ability to draw definitive causal inferences from the data. Unmeasured variables or reversed causation could influence the observed relationships, which requires caution in interpreting the directionality of effects. Future research could benefit from longitudinal designs to establish more robust causal pathways.

Second, the sample, comprising 263 academics, is relatively specific to the field of sports sciences within higher education, which may limit the generalizability of the findings to other academic disciplines or sectors. The unique organisational culture and work environment of sports science departments might have shaped the results in ways that are not fully representative of those in other fields. Broader sampling across diverse academic disciplines could provide a more comprehensive understanding of the interactions amongst these constructs.

Third, the study relied exclusively on self-report measures, which can introduce biases such as social desirability and standard method variance. Although steps were taken to ensure the reliability and validity of the instruments used, future studies might incorporate objective measures or multi-source data to mitigate these potential biases. Additionally, using self-reported scales for constructs like Psychological Safety and Job Satisfaction may not capture the full complexity of these experiences in the workplace.

Finally, cultural and institutional differences within the sample may have affected the findings. Most participants were from public institutions in Turkey, where organisational dynamics, job security, and administrative expectations might differ from those in other countries or private institutions. Consequently, future research should consider cross-cultural comparisons to better understand the contextual factors influencing these organisational dynamics.

Despite the contributions of this study, several limitations should be acknowledged. First, the data were collected using self-report measures, which may introduce response bias despite the use of Harman’s one-factor approach. Second, the cross-sectional design of the study limits the ability to draw causal conclusions amongst the examined variables. Future studies could employ longitudinal or experimental designs to better examine causal relationships. Additionally, because all variables were collected from the same respondents, the results may be affected by common method variance. Although established measurement scales and statistical procedures were used to mitigate this issue, future research may benefit from using multiple data sources or time-lagged research designs. Regarding the measurement model, all items from the original validated scales were retained as they demonstrated strong factor loadings and contributed to overall construct validity. However, it is important to note that reliance on self-report measures in sport science academia may be subject to social desirability bias. Future research could incorporate multi-source data to further validate these perceptions within the unique dual-role environment of academic sports faculties.

## 6 Practical implications and conclusion

The findings of this study have several practical implications for organisational leaders, administrators, and policymakers within higher education, particularly in sports sciences. The significant relationships identified amongst Organisational Commitment, Psychological Safety, Job Satisfaction, and Turnover Intention suggest that fostering a supportive, inclusive organisational environment is crucial for retaining talent and promoting well-being amongst academics.

First, the study underscores the importance of enhancing Organisational Commitment to improve Psychological Safety and Job Satisfaction. Institutions may consider implementing targeted interventions, such as leadership training, that promote inclusive and supportive management practises, fostering employee commitment and safety. This could involve creating clear communication channels, encouraging participatory decision-making, and providing regular feedback to ensure employees feel valued and heard. The positive impact of Psychological Safety on Mental Well-Being and Job Satisfaction also suggests that initiatives to improve interpersonal dynamics, such as team-building activities and conflict resolution workshops, could contribute to a more positive organisational climate.

Given the central role of organisational commitment in the present model, leadership and governance practises aimed at strengthening commitment appear particularly relevant. Transformational leadership, characterised by vision articulation, individualised consideration, and inspirational motivation, has been consistently associated with higher levels of affective commitment in academic and organisational settings (Mahfouz et al., 2020). Similarly, participative and transparent governance practises, including inclusive decision-making processes and clearly communicated promotion criteria, may foster stronger emotional attachment to the institution (Steffens et al., 2017). Policies that support professional development, mentoring systems, and recognition of academic contributions may further reinforce commitment-based bonds, thereby indirectly reducing turnover intentions.

Given that job satisfaction demonstrated the strongest direct negative effect on turnover intention in the present model, institutional strategies should prioritise structural factors influencing satisfaction. Balanced workload distribution, transparent promotion systems, equitable evaluation criteria, and work–life balance policies may represent more effective retention strategies than identity-enhancing initiatives alone.

From a practical perspective, the findings highlight the importance of fostering supportive organisational environments within sport science faculties. University administrators may benefit from implementing policies that strengthen organisational commitment and enhance job satisfaction, such as transparent promotion systems, fair workload distribution, and initiatives that support employee well-being. Creating psychologically safe academic environments where staff feel comfortable expressing ideas and concerns may also contribute to improved workplace experiences and reduced turnover intentions.

Second, although Organisational Identification was found to have a limited direct impact on Turnover Intention, its influence on Mental Well-Being suggests that organisational leaders should focus on strategies that promote a strong sense of belonging and purpose amongst employees. Such strategies include aligning organisational goals with personal values, providing opportunities for professional development, and recognising individual contributions to the organisation’s mission.

Finally, the study highlights the complex interplay of factors influencing Turnover Intention, suggesting that reducing turnover in higher education requires a multifaceted approach that addresses both intrinsic and extrinsic motivators. Institutions should strive to provide competitive salaries, job security, and career advancement opportunities whilst fostering an environment where employees feel psychologically safe and professionally fulfilled.

In conclusion, this study contributes to the growing literature on organisational dynamics in higher education by elucidating the direct and indirect relationships amongst key organisational variables. The findings emphasise the importance of creating supportive and committed organisational cultures to enhance job satisfaction, mental well-being, and employee retention. Future research should continue to explore these relationships across different contexts and employ longitudinal designs to build on the insights generated by this study.

## Data Availability

The raw data supporting the conclusions of this article will be made available by the authors, without undue reservation.
